# Untying the knot: Unraveling genetic mechanisms behind black knot disease resistance in *Prunus salicina* (Japanese plum)

**DOI:** 10.1002/pei3.70016

**Published:** 2024-11-05

**Authors:** Chloe Shum, Mohsen Najafabadi, Maxime de Ronne, Davoud Torkamaneh, Walid El Kayal, Jayasankar Subramanian

**Affiliations:** ^1^ Plant Agriculture, Ontario Agricultural College University of Guelph Guelph Ontario Canada; ^2^ Institut de Biologie Intégrative et Des Systèmes (IBIS) Université Laval Quebec City Québec Canada; ^3^ Faculty of Agricultural and Food Sciences American University of Beirut Beirut Lebanon

**Keywords:** antifungal, *Apiosporina morbosa*, genomics, GWAS, marker assisted breeding, *Prunus salicina*

## Abstract

Little is known regarding the genes, compounds and physiological alternations that take place upon infection of black knot disease. This research aimed to unravel the genetic mechanism responsible for the resistance of Japanese plum (*Prunus salicina* L.) trees against black knot (*Apiosporina morbosa* Schwein.) using a Genome‐Wide Association Study. Genotyping by Sequencing (GBS) was combined with a phenotyping system to analyze 200 genotypes of mixed origin. Population stratification identified four subpopulations, and the Fixed and Random Model Circulating Probability Unification (FarmCPU) algorithm was used for this analysis. Nineteen single nucleotide polymorphisms (SNPs) significantly associated with black knot disease resistance were discovered across five chromosomes. Linkage disequilibrium analysis identified 55 genes near these SNPs, with eight genes related to plant defense, immunity, and biotic stress response. One SNP mutation was found in the 5′ untranslated region of a gene regulating the first enzyme in phenylpropanoid biosynthesis. The results provide valuable insights into the genetic mechanisms behind BLACK KNOT disease resistance in Japanese plum and identifies potential markers for use in molecular breeding.

## 1 INTRODUCTION

Fungal diseases remain a serious threat to the global horticultural industry as the pathogens are constantly evolving to evade control (Keane, 2012). Only a few varieties of Japanese plum (*Prunus salicina* L.) are grown commercially but have limited resistance to fungi (Slingerland et al., 2022). This further enhances the spread of diseases, and often resistance is broken in these varieties.

Black knot disease, caused by the fungal pathogen *Apiosporina morbosa* (Schwein.) represents a significant threat to plum production. As the name suggests, the disease manifests as dark, conspicuous, woody swellings or galls along the branches of infected trees, ultimately leading to reduced yield, dieback and in severe cases, tree mortality (Koch, 1933; Zhang et al., 2005).

The only measurable symptom of black knot infection is the presence of the knots themselves. A strong correlation exists between the size and number of knots, and the severity of infection. Since the fungus does not affect the leaves, fruit, or any other visible parts of the tree aside from the branches, no additional physiological markers are available for assessment (Wilcox, 1992). Black knot infection occur via two primary modes, the first involves the germination of ascospores on the epidermis of young shoots or exposed cambium (Koch, 1935), and the second involves hyphae and conidia spreading internally from existing knots to other areas (El Kayal et al., 2021). As infection intensifies, additional symptoms such as girdling and leaf drop will emerge (Wisconsin Horticulture, n.d.). The knot itself is a combination of fungal tissue and the tree's attempt at compartmentalization through surrounding the pathogen with callus tissue (Moorman, 2023). The hypertrophy of the tissue is commonly seen in canker‐causing diseases and leads to the perennial growth of the knots (Smith, 1970).

The economic impact of black knot disease in plum orchards necessitates the development of effective disease management strategies, with genetic resistance emerging as a promising avenue for sustainable control. Two fifths of variable costs in plum production are spent on pruning and fungicides in Canada (Ontario Ministry of Agriculture, Food and Rural Affairs, 2023).

With the plum market expanding in North America, it is imperative that black knot is managed efficiently, as it hinders production (FAOSTAT, 2024). Chemical treatments have shown to be ineffective long term and unworthy of the investment since outbreaks vary from year to year (McFadden‐Smith et al., 2000). Furthermore, wild *Prunus* in adjacent forests can be sources of inoculum (Douglas, 2008).

Black knot has long posed many challenges for researchers, and thus has been understudied. Over 24 species of both wild and cultivated *Prunus* are hosts of black knot (Snover & Arneson, 2002). The difficulty in studying *A. morbosa* arises from its complex life cycle. Infections often go unnoticed during the early stages, as the galls can take over a year to become visible (Koch, 1935). This delayed symptom expression complicates early detection and timely intervention. The activation of spore release has a wide range of acceptable conditions, as dew alone has been enough to prompt it (McFadden‐Smith et al., 2000). The demanding conditions and the long duration of disease development makes it almost impossible to do any in vitro assay for testing against black knot resistance. Developing varieties of plums which are resistant to black knot would be an ideal outcome, but due to lengthy maturation periods until fruit set, a breeding cycle can take 18–20 years (Eremin et al., 2022).

A genome‐wide association study (GWAS) will leverage bioinformatic algorithms that can process large amounts of genomic data on many individuals. We chose to do a GWAS as it enables us to develop genomic markers from our diverse population of Japanese plum. The result is a filtered view of the entire genome, pointing to loci of interest which are associated with the trait. With this knowledge, breeders can ensure that progeny contain these markers early on in the process through genotyping. Using accelerated breeding methods provides the plum industry with a cost‐effective and environmentally favorable way of suppressing this plague.

## 2 MATERIALS AND METHODS

### 2.1 Plant materials and phenotyping

A total of 200 genotypes of Japanese plum (*Prunus salicina* L.) grown at Vineland Research Station constituted the plant materials for this study. For each accession, five leaves were taken and lyophilized using a bulk tray dryer (Labconco, Kansas City, MO, USA) and stored. Approximately 100 mg of leaf tissue was placed in a microcentrifuge tube with glass beads to undergo milling in a Mixer Mill MM 400 (Retsch, Haan, Germany). DNA extraction from the lyophilized leaf samples was carried out using Norgen Plant and Fungi DNA Extraction Kit (Norgen Biotek Corp., Thorold, ON, Canada) following their guidelines. DNA quantification was tested on a Nanodrop Spectrophotometer (Thermo Fisher Scienfitic, Walham, MA, USA) to ensure the quality of DNA.

**FIGURE 1:**
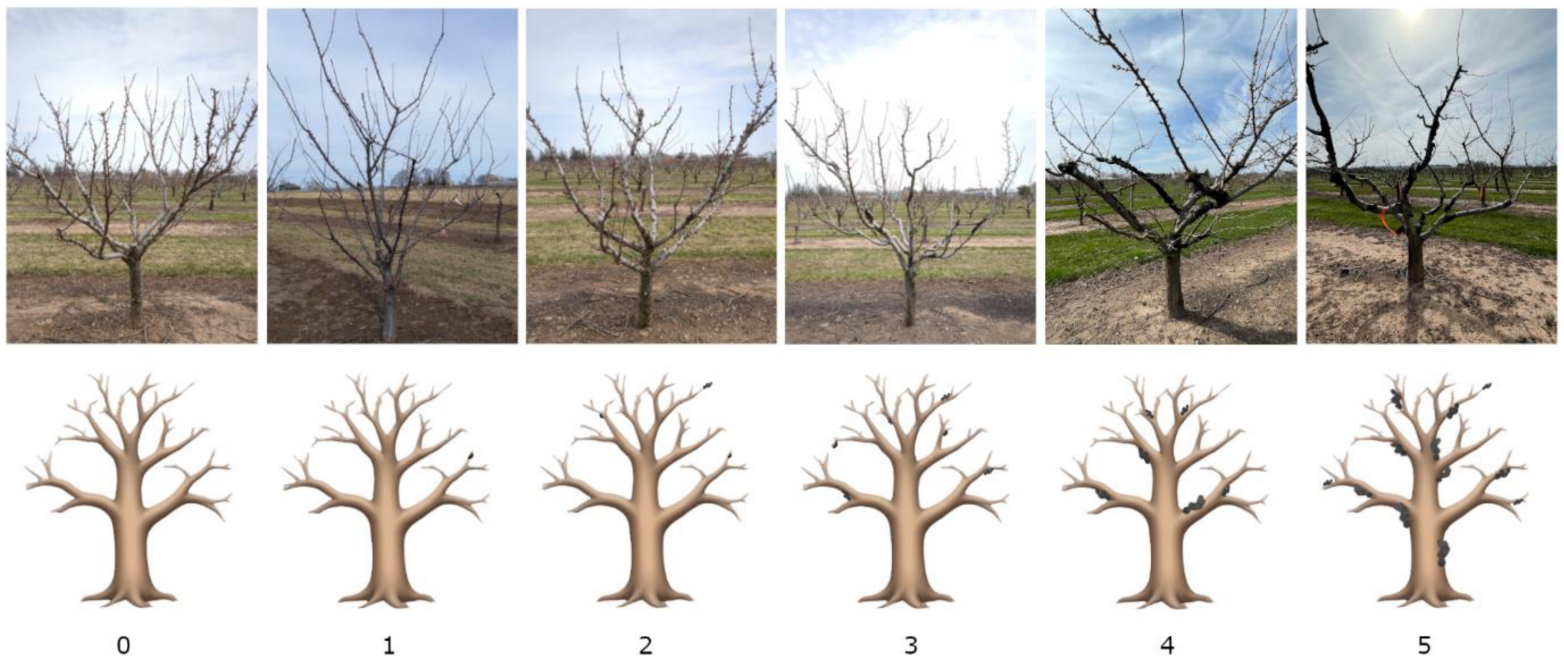
Disease severity scale (DSR) of black knot disease in Japanese plum (*Prunus salicina* L.). Above are photos taken from Vineland Station, but the nature of the disease makes capturing the entire state of the tree difficult, thus an animation has been included to further aid visualization. Photo credit: (Bogosvayatska, n.d.) *A bare tree without leaves*. Adapted from https://emojis.sh.

Every tree in the sampled population was phenotyped 4 times by multiple people on separate days. This design helped to reduce any variability between the subjective ratings that could have resulted from misjudgment due to a myriad of factors such as weather, visibility issues etc. The disease severity rating (DSR) was ranked from 0 to 5 based on the number and size of knots and the general health of the tree which is an improved rating system based on Shinde et al. (2024). In brief, a rating of 0 indicates a healthy black knot free tree while a rating of 5 signifies a severely infested tree with knots of various sizes covering the majority of the branches and deep in the scaffolds (Figure 1). As the trees progress in disease severity, they also exhibit diminishing health and often dead branches. Since this was a visual rating system based on subjective analysis of each rater, a calibration step was done to minimize the subjective variability for the tested cohort of trees. The median was taken from the four sets of ratings. The histograms to plot the distribution of DSR were done in R v4.1.1 with the ‘*hist*’ function (R Core Team, 2021).

### 2.2 Library preparation and sequencing

DNA samples were adjusted to a final concentration of 10 ng/μL and prepared in 96 well plates. Sequencing was performed using an Illumina NovaSeq 6000 platform (Illumina, CA, USA), with paired‐end reads generated with a length of 150 base pairs (bp). The sequencing service was provided by Genome Quebec Service and Expertise Center (CESGQ), (Montreal, QC, Canada). Library preparation adhered to the ApeKI restriction enzyme protocol as described by Kaur et al. (2017).

### 2.3 SNP calling and filtration

The reference genome assembly used for aligning genotyping by sequencing (GBS) reads and calling single nucleotide polymorphisms (SNPs) was from the cultivar ‘Sanyueli’ of *P. salicina* retrieved from the National Center for Biotechnology Information (NCBI) database under the accession number GCA_014863905.1_SCAU_Psal_1.0_genomic. SNPs were called using the Fast‐GBS pipeline developed by Torkamaneh et al. (2020). It involved aligning GBS reads to the reference genome assembly and identifying positions where sequence variations occurred, indicative of potential SNP loci. The variants were then processed by Platypus to improve specificity of calls (Rimmer et al., 2014). Quality control measures were implemented using VCFtools v0.1.16, to exclude variants with >80% missing data and low‐quality scores (QUAL <10 and MQ <30) (Danecek et al., 2011). Imputation of missing SNPs was carried out using BEAGLE 4.1 (University of Washington, Seatle, WA, USA), followed by removing SNPs with heterozygosity less than 50% and a minor allele frequency (MAF) of <0.05. A minor allele count (MAC) of 5 was selected, and only biallelic SNPs were retained for further analysis. This filtration process yielded 20,579 SNPs, which were subsequently utilized for population structure and GWAS analyses.

## 3 STATISTICAL ANALYSIS FOR GWAS

### 3.1 Population structure analysis

Initially, a total of 20,579 high‐quality SNPs from the tested population were used to conduct the population structure analyses using the STRUCTURE software (Pritchard et al., 2000). Five runs were conducted for K set from 1 to 10 to estimate the most appropriate number of subpopulations by using the K tool from the fastSTRUCTURE software. In order to better estimate the genomic relationships among genotypes, TASSEL5 (Cornell University, New York USA) was used to compile the kinship matrix, which was then plotted into a heatmap in R using the ‘*heatmap*’ function (R Core Team, 2021).

### 3.2 Linkage disequilibrium decay (LD) estimation

Following population structure analysis, LD was determined using all the SNP profiles of the tested population. Pairwise‐LD table was calculated in TASSEL and the curve was generated following Remington et al. (2001) in R.

### 3.3 GWAS model

The GWAS model Fixed and random model Circulating Probability Unification (FarmCPU) was used to comprehensively interrogate genotype–phenotype associations using an integrated R package rMVP (Memory‐Efficient, Visualize‐Enhanced, Parallel‐Accelerated GWAS) (Yin et al., 2021). FarmCPU leverages the computational capabilities of both mixed linear model and fixed effect models. It is a multi‐locus GWAS method, enabling the identification of additional SNP‐trait associations by explicitly modeling and correcting for genetic relatedness (Liu et al., 2016).

### 3.4 Quantile‐quantile (Q‐Q) plots

For each GWAS, a corresponding Q‐Q plots was generated. These plots provided visual assessments of the observed versus expected distribution of test statistics, offering insights into potential inflation or deflation of associations.

## 4 CANDIDATE GENE IDENTIFICATION

**FIGURE 2:**
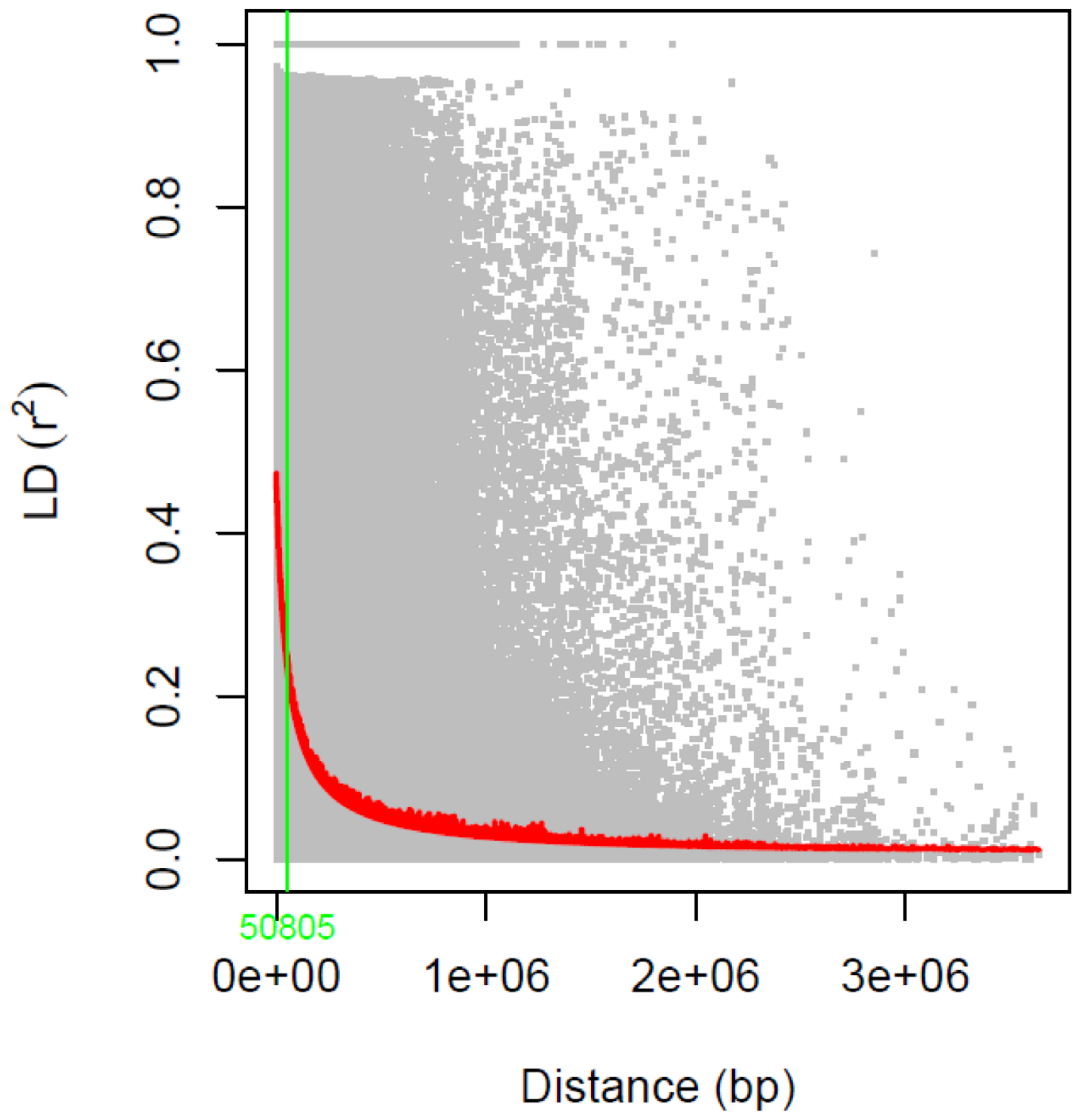
Scatterplot of genome‐wide linkage disequilibrium (LD) decay based on 20,579 SNPs. Dots show the pairwise LD measurements between two markers at a given distance having minor allele frequency (MAF) ≥ 0.05. The red line represents nonlinear function of r^2^ pertaining to a physical distance (bp). Green vertical line indicates the genetic distance where the r^2^ value is reduced by half.

The result from the LD analysis was used for gene discovery. According to the LD analysis, the distance in which the *r* ^2^ value was 50% of the initial was at 50805 bp (Figure 2). A window of 25 K bp below and above a SNP was investigated. All genes falling within this window were included in the preliminary analyses. The BLAST annotations from these genes were considered based on their descriptions provided by the Rosaceae Gene Database (Jung et al., 2019).

## 5 RESULTS

### 5.1 Population structure and kinship

**FIGURE 3:**
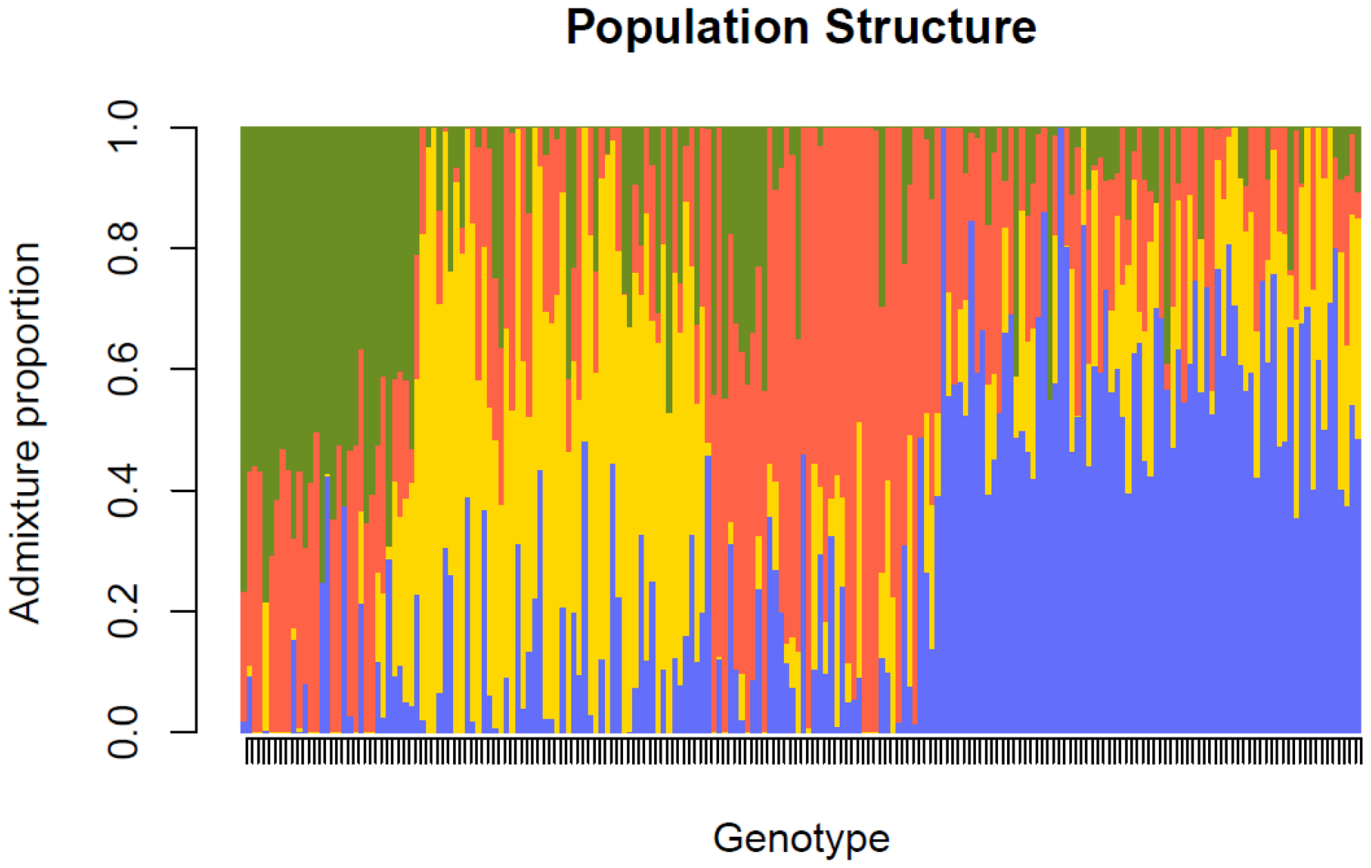
Population structure based on ADMIXTURE analysis according to STRUCTURE software (Pritchard et al., 2000), subpopulations (K) = 4. Plot was derived from 200 genotypes of Japanese plum (*Prunus salicina*), ordered by q values (ancestry proportions). Each genotype is represented by an individual vertical line, separated into colors that represent the probability of that individual belonging to a subpopulation.

**FIGURE 4:**
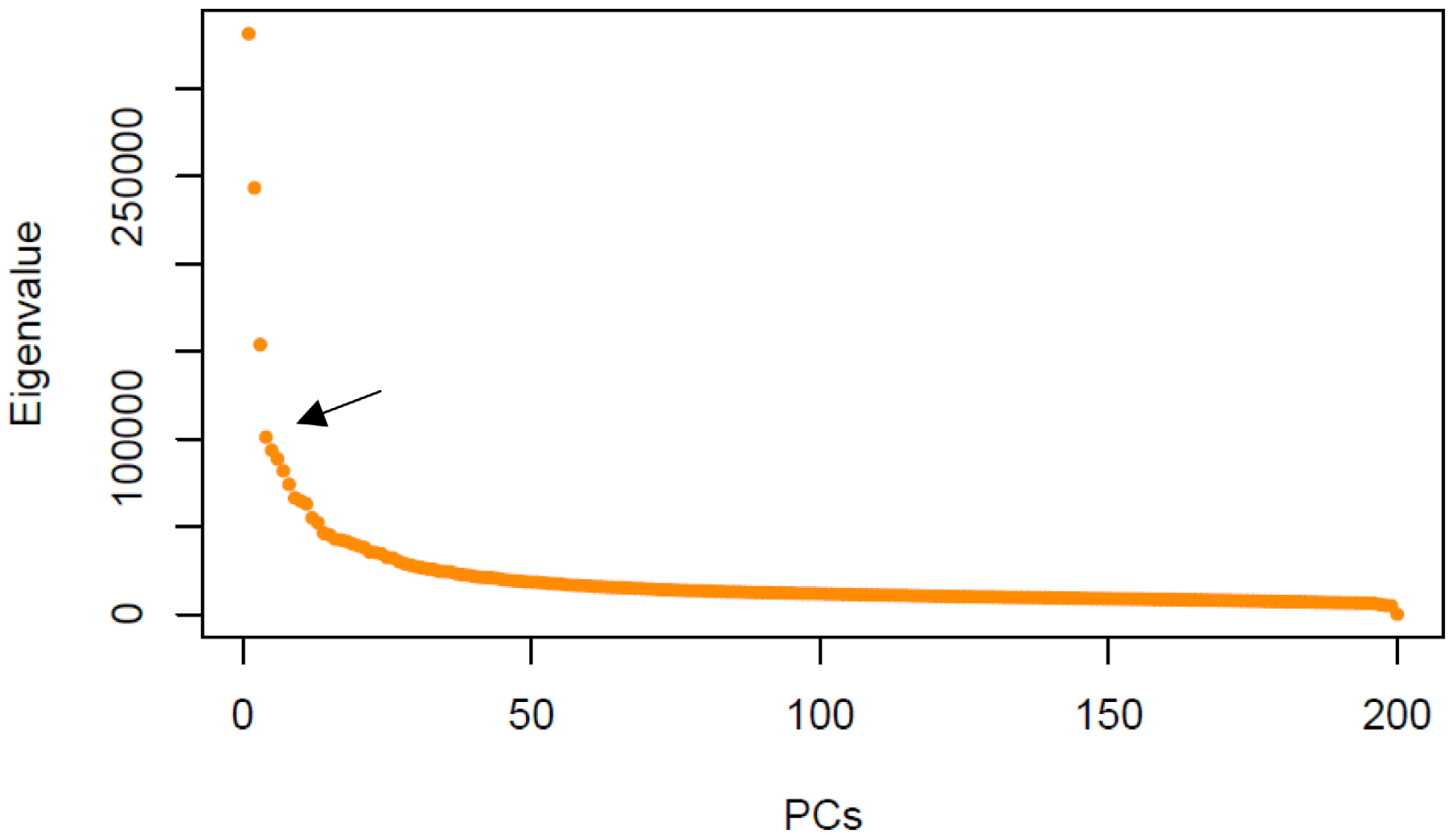
Scree plot showing the amount of variance, depicted by number of eigenvalues, based on single nucleotide polymorphism (SNP) data from genome‐wide association study (GWAS). The y‐axis represents the eigenvalues, while the x axis represents each principal component (PC). The optimal number can be seen as 4 as pointed out by the arrow, since points level off afterwards, indicating diminishing variance explained with each subsequence PC.

**FIGURE 5:**
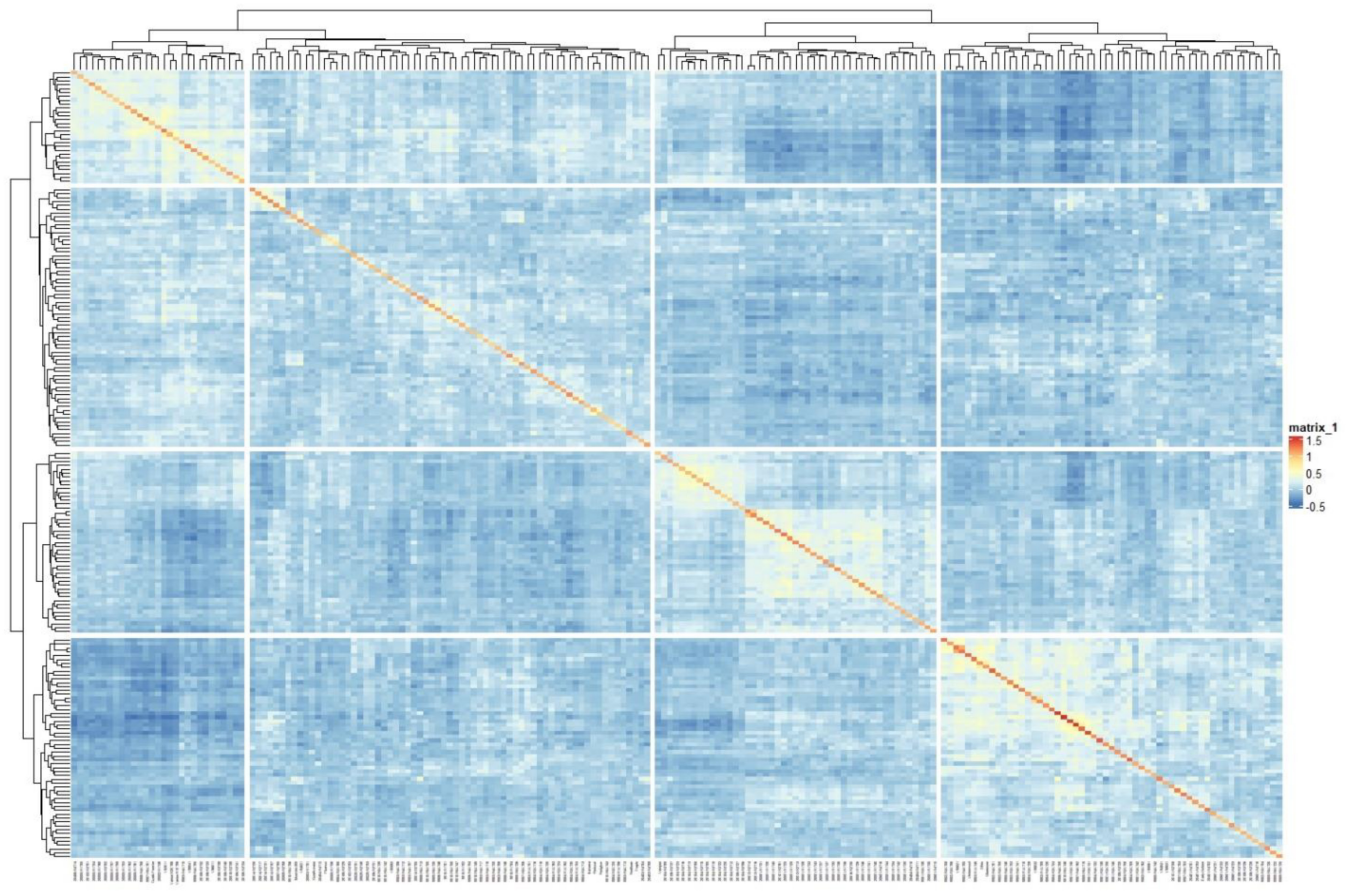
A phylogenetic tree based on a kinship matrix derived from single nucleotide polymorphism (SNP) data. The heat map shows the degree of relatedness between paired genotypes. The level of relatedness based on scale to the right, the warmer the color, the higher degree of relation. The population is separated into four main subpopulations. Names of the genotypes are on the x‐axis.

**FIGURE 6:**
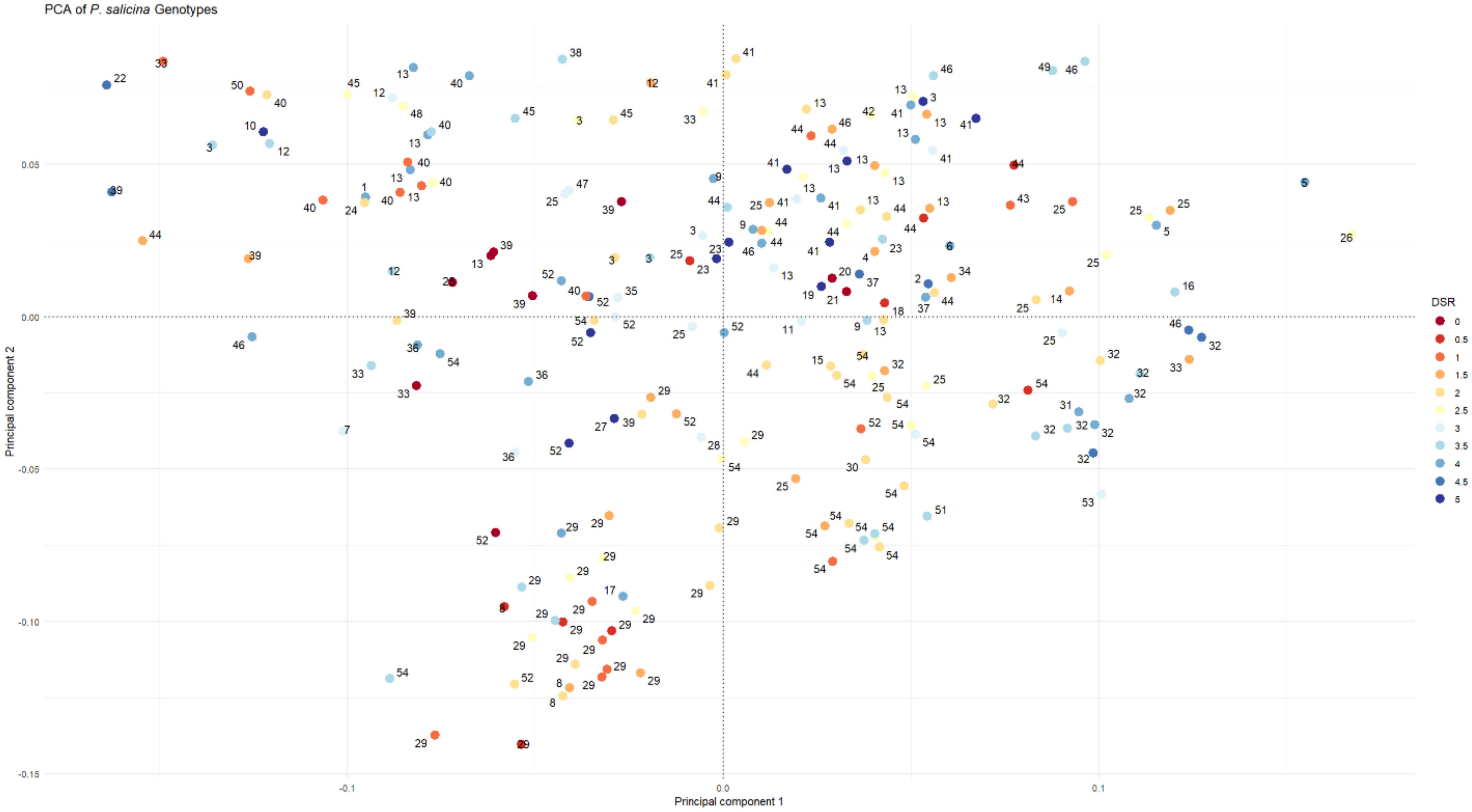
PCA (principal component analysis) plot of single nucleotide polymorphism (SNP) data from 200 individuals mapped on principal component 1 (x‐axis) and principal component 2 (y‐axis) generated from a pairwise distance matrix. Numbers represent the parental crosses (list available in Table S1). Color of dots denote the phenotypic disease severity rating (DSR), the warmer the color, the higher the rate of infection of a genotype.

The population structure analyses yielded an optimal 4 subpopulations (Figure 3). According to the scree plot (Figure 4), any principal component (PC) beyond 4 did not provide any significant increase in explanation of variance. The phylogenetic tree (Figure 5) showed four distinct groups which may be a result of the sample population containing many accessions which had parent and sibling relationships. The analysis prevented the misinterpretation of phenotype‐marker associations which could have led to false positives. As expected, genotypes which shared parents typically appeared closer together in the PCA (Figure 6). Further GWAS analyses were based on *K* = 4.

### 5.2 Phenotypic evaluations

**FIGURE 7:**
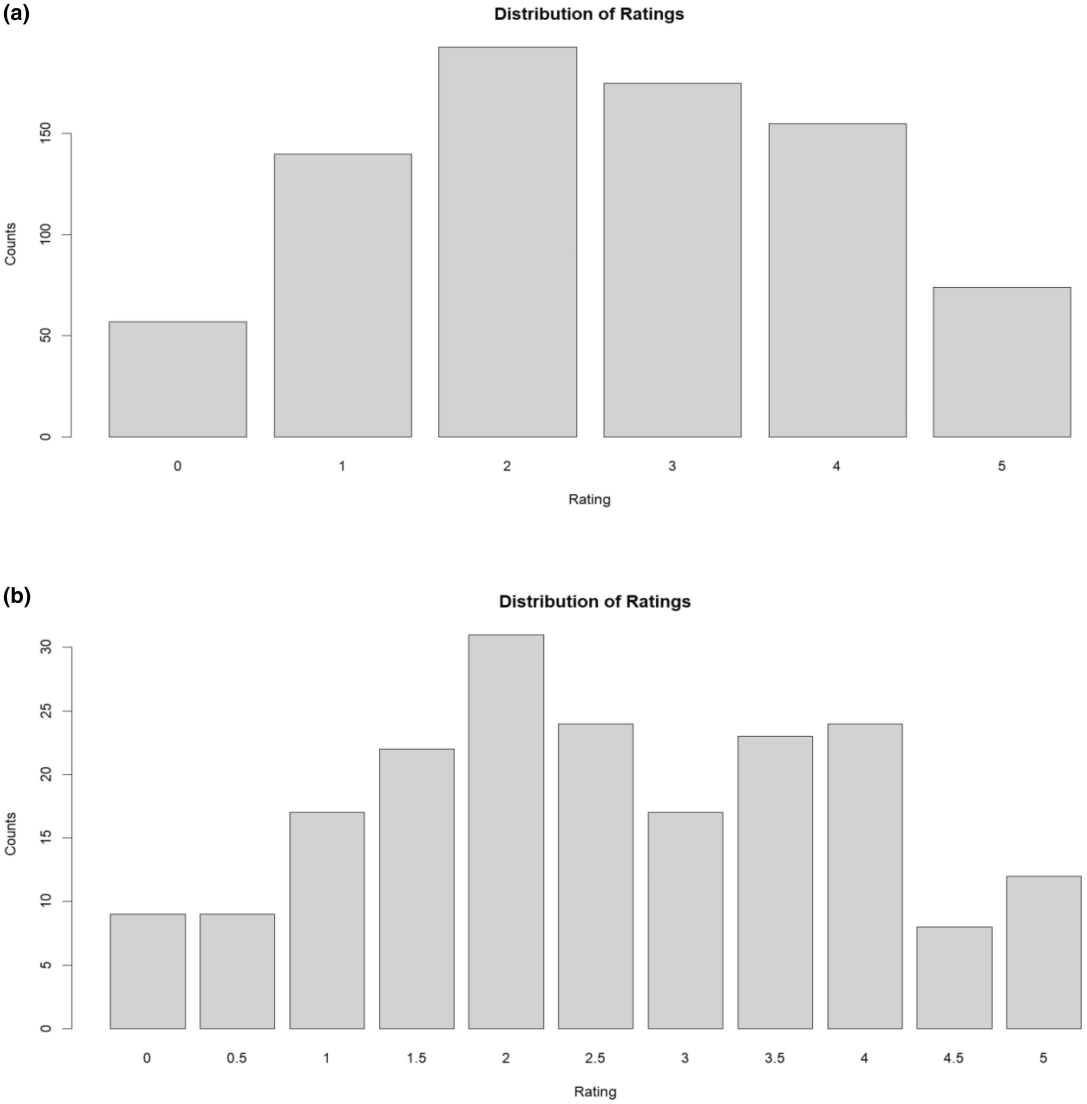
Bar plots of Disease Severity Ratings (DSR) for 200 individuals of Japanese plum (*Prunus salicina* L.). (a) Total raw counts of the DSR from two independent raters on two separate days (b) Counts of the DSR after calculating and including the median of the raw counts.

A median of the four ratings was generated, so that in total there were 5 datasets to run GWAS analyses on. Since the population size (*n* = 200) was relatively small for a GWAS, having more abundant phenotypic data was a way to offset this limitation. The raw counts (Figure 7a) showed that the counts were highest for rating 2, followed by 3, 4, 1, 5 then 0. The most extreme ratings, 0 and 5 contained the fewest genotypes, while 2 and 3 held similarly high ratings. After taking the median of the four data sets (Figure 7b), additional bins containing 0.5 increments were generated, thus increasing the number of bins from 6 to 11. Similarly, the ratings on both sides of the extremes, 0, 0.5, 4.5 and 5 had the fewest counts. Rating 2 still presented the highest number of counts, followed by 2.5 and 4. Rating 3.5 followed closely, then by 1.5, and by 1 and 3 equally. The additional bins were crucial for improving the resolution between 0 and 1, this being the most important distinction in phenotyping. As a rating of 0 was the lower boundary and entailed no sign of infection, it should in essence never have been rated any higher. When the median of all the raw counts was taken, the trees which were sometimes given a higher rating would have been reclassified as a 0.5 instead of a 0. This removed false negatives, as we could see the number of trees rated zero in the raw counts dropped from7% compared to 4.5% after the median was calculated.

### 5.3 Genotypic evaluations

High quality SNPs from paired‐end 150 bp ion torrent reads were generated for the GWAS using Fast‐GBS v2.0 (Torkamaneh et al., 2020). The number of raw SNPs generated was 515,551. 335,650 SNPs were low quality and removed, leaving behind 179,861 SNPs. After removing any SNPs in the scaffolds, SNPs appearing fewer than 4 times in all lines, SNPs missing greater than 80% of the calls, and a MAF of 5%, there were 30,637 remaining. A total of 20,579 SNPs were retrieved post‐imputation filtration. Across all 5 GWAS runs, FarmCPU consistently resulted in the differentiation of significant SNPs compared to other algorithms (Liu et al., 2016). A total of 15 SNPs met the significance threshold. SNPs were found on chromosomes 1, 2, 4,6, 7 and 8 (Table 1). SNP16912 was discovered in most GWAS analyses, suggesting a potential link to black knot resistance. Likewise, SNP20606, SNP341, SNP10301 and SNP 5103 also appeared in multiple GWAS analyses.

**TABLE 1:** Results of the single nucleotide polymorphism (SNP) BLAST search through the Rosaceae.org database. SNPs identified from the genome‐wide association study (GWAS) were queried with a search window of ±25,000 base pairs, as informed by linkage disequilibrium (LD) decay analysis.

Chromosome	Start position	Stop position	SNP ID	Ortholog	BLAST
Chr1	4,324,219	4,327,831	341	AT4G13460.2	SU(VAR)3–9 homolog 9
Chr1	4,316,774	4,320,032	341	AT1G04555.1	Transmembrane protein
Chr1	4,314,023	4,314,811	341	AT1G48520.2	GLU‐ADT subunit B
Chr1	4,310,816	4,313,810	341	AT1G48520.1	GLU‐ADT subunit B
Chr1	4,308,428	4,309,687	341	AT4G17565.1	F‐box protein (DUF295)
Chr1	4,305,492	4,306,346	341	AT4G13450.1	Adenine nucleotide alpha hydrolases‐like superfamily protein
Chr1	4,339,649	4,340,728	341	AT5G05210.2	Surfeit locus protein 6
Chr1	4,337,964	4,338,920	341	AT1G04560.1	AWPM‐19‐like family protein
Chr1	46,164,514	46,165,051	3785	PRUPE_1G498700	Hypothetical protein
Chr1	46,156,492	46,157,089	3785	PRUPE_1G498600	Hypothetical protein PRUPE
Chr1	46,143,395	46,145,368	3785	AT1G21830.1	AT1G21830.1
Chr1	46,138,847	46,142,212	3785	AT1G42550.1	Plastid movement impaired1
Chr1	46,136,030	46,137,281	3785	A0A4Y1RUX2_PRUDU	Uncharacterized protein
Chr2	18,728,368	18,732,248	5103	AT3G57810.4	Cysteine proteinases superfamily protein
Chr2	18,742,498	18,745,025	5103	A0A5H2Y143_PRUDU	Retinoblastoma‐related protein‐like
Chr2	35,153,758	35,157,921	6558	AT4G25340.1	FK506 BINDING PROTEIN 53
Chr2	35,160,083	35,164,374	6558	AT2G36530.1	Enolase
Chr2	35,165,258	35,167,019	6558	AT3G22660.1	rrna processing protein‐like protein
Chr2	35,167,807	35,168,633	6558	AT5G07165.1	Transmembrane protein
Chr2	35,169,528	35,170,248	6558	PRUPE_2G282200	Hypothetical protein
Chr2	35,170,302	35,174,379	6558	AT4G25320.1	AT hook motif DNA‐binding family protein
Chr2	35,174,675	35,180,105	6558	AT5G51600.1	Microtubule associated protein (MAP65/ASE1) family protein
Chr4	6,143,771	6,153,332	10,301	AT5G12170.2	CRT (chloroquine‐resistance transporter)‐like transporter 3
Chr6	24,269,465	24,275,036	15,122	AT5G10200.3	ARM‐repeat/Tetratricopeptide repeat (TPR)‐like protein
Chr6	24,279,521	24,283,387	15,122	A0A314XM19_PRUYE	Cysteine‐rich and transmembrane domain‐containing protein WIH1‐like
Chr6	24,285,690	24,286,288	15,122	AT2G32190.1	Cysteine‐rich/transmembrane domain A‐like protein
Chr6	24,298,690	24,301,642	15,122	AT3G61130.1	Galacturonosyltransferase (GAUT) 1
Chr6	34,935,856	34,937,988	16,336	AT4G14746.1	Neurogenic locus notch‐like protein
Chr6	34,938,912	34,940,971	16,336	AT3G04790.1	Ribose 5‐phosphate isomerase%2C type A protein
Chr6	34,941,945	34,945,097	16,336	AT3G04780.1	PITH domain protein (DUF1000)
Chr6	34,945,281	34,950,366	16,336	AT5G49570.1	Peptide‐N‐glycanase 1
Chr6	34,953,026	34,955,743	16,336	AT4G29250.1	HXXXD‐type acyl‐transferase family protein
Chr6	34,957,805	34,958,865	16,336	A0A4Y1RSD7_PRUDU	NAD(P)‐linked oxidoreductase superfamily protein
Chr6	34,971,434	34,973,382	16,336	AT5G44500.2	Small nuclear ribonucleoprotein family protein
Chr7	10,707,937	10,720,569	16,912	AT1G25270.1	Nodulin MtN21 /EamA‐like transporter family protein
Chr7	10,720,870	10,721,789	16,912	AT1G68170.1	Nodulin MtN21 /EamA‐like transporter family protein
Chr7	16,488,569	16,489,126	17,217	AT5G26330.1	Cupredoxin superfamily protein
Chr7	16,489,425	16,491,854	17,217	AT5G06660.1	Transmembrane/coiled‐coil protein
Chr7	16,495,743	16,496,570	17,217	AT1G67030.1	Zinc finger protein 6
Chr7	16,505,663	16,516,322	17,217	AT3G12020.4	P‐loop containing nucleoside triphosphate hydrolases superfamily protein
Chr8	26,057,477	26,057,877	20,380	A0A251N2H9_PRUPE	Major pollen allergen Ole e 6‐like
Chr8	26,058,609	26,059,443	20,380	AT2G48140.1	Bifunctional inhibitor/lipid‐transfer protein/seed storage 2S albumin superfamily protein
Chr8	26,060,079	26,061,239	20,380	AT3G22600.1	Bifunctional inhibitor/lipid‐transfer protein/seed storage 2S albumin superfamily protein
Chr8	26,062,926	26,063,918	20,380	AT4G14815.1	Bifunctional inhibitor/lipid‐transfer protein/seed storage 2S albumin superfamily protein
Chr8	26,067,649	26,071,961	20,380	AT3G63220.3	Galactose oxidase/kelch repeat superfamily protei
Chr8	26,072,395	26,075,801	20,380	AT1G03110.1	Transducin/WD40 repeat‐like superfamily protein
Chr8	26,080,145	26,084,474	20,380	AT3G22550.1	NAD(P)H‐quinone oxidoreductase subunit%2C putative
Chr8	27,536,014	27,544,150	20,606	AT3G02680.1	Nijmegen breakage syndrome 1
Chr8	27,542,189	27,544,150	20,606	AT1G55865.1	Ubiquitin‐protein ligase
Chr8	27,544,287	27,545,236	20,606	AT3G36659.1	Plant invertase/pectin methylesterase inhibitor superfamily protein
Chr8	27,545,607	27,549,021	20,606	AT2G47490.1	NAD+ transporter 1
Chr8	27,549,339	27,553,073	20,606	AT4G12760.1	RPA‐interacting protein A
Chr8	27,521,637	27,524,173	20,606	AT4G16110.1	Response regulator 2
Chr8	27,525,058	27,527,972	20,606	AT4G02210.2	myb/sant‐like dna‐binding domain protein
Chr8	27,529,926	27,533,650	20,606	AT3G02690.1	Nodulin mtn21 /eama‐like transporter family protein
Chr8	27,534,548	27,535,995	20,606	AT1G65690.1	Late embryogenesis abundant (LEA) hydroxyproline‐rich glycoprotein family

### 5.4 Candidate gene identification

In total, 55 putative genes were uncovered within the 25 K bp genomic regions flanking the 15 SNPs of interest. They were discovered through the Rosaceae Database which cataloged all of the known genes in the regions searched, including homologous genes in a closely related species such as peach (*Prunus persica* L.), as it is a more highly annotated species. Eight of these genes had links to plant immunity and defense according to their BLAST annotations.

Of the eight genes, most only had precise annotations from the *Arabidopsis thaliana* (L.) Heynh genome. SNP15122 had the most numerous links to genes, *AT5G10200.3* an ARM‐repeat/Tetratricopeptide repeat (TPR)‐like protein, *AT2G32190.1* a cysteine‐rich/transmembrane domain A‐like protein and *AT3G61130.1* galacturonosyltransferase (*GAUT*) 1. These proteins are involved in hormone signaling, auxin distribution, and cell wall biosynthesis respectively. SNP5103 along with SNP17217 were linked to *AT3G57810.4* a cysteine proteinases superfamily protein and *AT1G67030.1* a zinc finger protein 6, which were involved in programmed cell death (PCD) upon pathogenic attack. SNP6558 was associated to *AT3G36659.1* which was a plant invertase and pectin methylesterase inhibitor. SNP10301 had also appeared twice in the GWAS analyses, and was associated with the gene *AT5G12170.2*, a chloroquine‐resistance transporter (*CRT*)‐like transporter 3 involved in glutathione homeostasis and stress response. SNP20606 was correlated to the *AT3G02690.1* gene, which was a nodulin MtN21 /EamA‐like transporter family protein.

Only one SNP was found within a genic region, SNP20380 on chromosome 8 fell in the 5′ untranslated region (UTR) of a Kelch repeat F‐Box protein, KFB20. It was a major negative regulator of cytokinin and phenylalanine ammonia‐lyase (*PAL*), the latter being the initial enzyme in the phenylpropanoid and lignin biosynthesis pathway (Kurepa et al., 2018; Zhang et al., 2013). This pathway was noted as significant in previous studies which identified phenolic compounds in resistant genotypes.

## 6 DISCUSSION

The goal of this research was to develop a deeper understanding of black knot resistance in Japanese plum. We have previously discovered a significant hormonal component to resistance (Shinde et al., 2023, 2024) which gave the current work some insights as to what mechanisms could be potentially involved since it had revealed hormonal alterations, resistance (R) genes and pathogenicity‐related genes. It is believed that resistance is polygenic, with multiple genes with various effect sizes determining the phenotype (Poland et al., 2009). Lacking a priori information of the inheritance patterns, genomic locations, or gene pathways involved, GWAS was the best approach.

Similar to the development of germplasm for breeding programs, an accurate GWAS requires precise and definitive characterization of the varieties. Thus, understanding relationships between genotypes is crucial, particularly in cultivated varieties, as it helps account for potential confounding factors. The trait we are analyzing, black knot resistance, is not a simple continuous trait. It is rather a categorical ordinal trait, and hence it was expected that the Q‐Q plot may have some abnormalities. Furthermore, it seems that the phenotype is highly related to the genetic background, resulting in many signals with extremely low *p*‐values. This abundance of low *p*‐values may indicate potential issues such as population stratification or other confounding factors, which can lead to inflation in test statistics. Even adjusting for population stratification and multiple testing corrections could not fully ameliorate this effect. One potential cause could be cryptic relatedness that population stratification and kinship analysis were unable to account for (Holland et al., 2017). Having a larger sample size could be a way to improve the results.

Additionally, there could be spurious associations from environmental factors that could not be accounted for in the calculations. Most trees had been planted over two decades ago, without the intention of studying black knot disease. Variations in the management practices during different periods could have positive/negative effects on black knot incidence. Differences in climate conditions would have been negligible, however more localized variables such as waterlogging in some years, tree spacing etc. could predispose the trees to sustained infections. We made sure not to selectively prune the knots for 10 years (as is the customary practice to control black knot) to avoid altering the DSR, but rather to prune all branches uniformly, diseased or healthy. However, adherence to these instructions may not have been consistent over the years.

Another explanation is that disease resistance is a good candidate to be an omnigenic trait, or a hyper‐polygenic trait. Through our GWAS, we found that many SNPs seemed to influence the genotype's ability to control black knot infection. According to the omnigenic model, complex traits are accumulations of gene expression effects from the entire genome in relevant tissues (Boyle et al., 2017). Changes in genes related to cell wall fortification, counteracting reactive oxygen species (ROS) damage, sucrose transport, and PCD can alter the resistance phenotype, albeit not directly. Within this framework, core genes include R genes, PR genes, and pattern recognition receptor (PRR) genes, as well as pathways for known secondary metabolite synthesis. Peripheral genes, which indirectly affect resistance to black knot virulence, include those related to cell wall structure, sugar transport, lignin biosynthesis, hormone pathways, epigenetic regulators, and transcription factors. *P. salicina* has a relatively small genome, 248 mb compared to other perennial fruit crops (Liu et al., 2020).

Based on our earlier studies, certain patterns can be ascertained related to the pathology and the host‐pathogen interaction (El Kayal et al., 2021; Shinde et al., 2023, 2024). First, the fungus causes the infected tissue to become corky, swollen blisters that are black in color. This is likely due to an altered synthesis of cytokinins and auxins (Shinde et al., 2023), as well as the production of allomelanin (Singla et al., 2021). Genes related to pathogen attack, hormonal signaling, and PCD were considered as functional candidate genes which are involved in the collective response towards black knot resistance. The linked gene *AT3G57810.4* to SNP5103 falls within the large cysteine proteinases superfamily, which is responsible for the plant's adaptive and innate immunity and invoking regulated cell death when necessary (Kopitar‐Jerala, 2012). *AT3G02690.1* is a nodulin MtN21/EamA‐like transporter family protein, these proteins are also known to enhance auxin and sucrose transport (Denancé et al., 2014). From observations by El Kayal et al. (2021), sucrose transport is heavily restricted in susceptible genotypes of plum.

The cell wall is both a structural and biochemical opposition against pathogens (Gigli‐Bisceglia et al., 2020). It is the first step where a pathogen interacts with the cell, and thus prevents a majority of infiltration. Pectin, one of the main components of the plant cell wall, is synthesized by the *GAUT* family of enzymes (Shin et al., 2021). The analysis identified a correlation with the gene *AT3G61130.1*, also known as *GAUT1*. Weaker pectin bonds can facilitate the infiltration of the fungus, while more rigid cell walls can impede its growth (Benoit et al., 2012).

Although little is known about the exact infection mechanism of *A. morbosa*, no haustoria or other penetration mechanisms were observed, instead clear fungal strands were seen growing through the cambium and past the xylem (Wainwright, 1970). The fungal strands were irregular in shape, as they assumed the shape in between the host cells (Wainwright, 1970). This type of interaction must invoke receptors on the cell wall and activate defenses.

Interestingly, *AT3G36659.1*, an invertases/pectin methylesterase inhibitor protein, was also found to be related. It's function is to counteract the activity of pectin degrading enzymes, which are abundant during fungal invasion. In *A. thaliana*, it has been shown to aid in defense against *Psudemonas syringae* Van Hall (Bethke et al., 2014) and *Botrytis cinerea* Pers. (Del Corpo et al., 2020). Pectin methylesterases (PMEs) are regulated by their inhibitors (PMEIs) and this system is vital in maintaining the structural and physiological properties of the cell wall. These proteins are capable of responding to pathogenic attack by fortifying the cell wall, changing elasticity, adhesion properties and porosity (Wormit & Usadel, 2018). A dysfunction in the inhibitors could lead to overactivity, advancing pectin degradation and enhancing susceptibility to disease (Del Corpo et al., 2020). Conversely, plants with overactive PMEIs may have greater inhibition towards PMEs of a pathogen. Overexpression of PMEI in *A. thaliana* increased methylesterification of the cell wall which led to higher resistance against fungal and bacterial nectrotrophs (Lionetti et al., 2007). Furthermore, in pepper (*Capsicum annum* L.), Ca*PMEI1*, was also found to be effective against *Fusarium oxysporum* f.sp. *matthiolae* (Baker) and *Alternia brassicicola* (Schwein.) in vitro (An et al., 2008). Therefore, PMEIs play a major role in microbial defense as they prevent degradation, while also acting antagonistically to invading pathogens.

In peach infected by *Lapiodiplodia theobromae* (Pat.) Griffon & Maubl, PMEI genes were upregulated (Gao et al., 2016). Additionally, six genes in the phenylpropanoid pathway were highly expressed in the infected versus the control. *L. theobromae* is a fungus that causes lesions in shoots. The symptoms include gummosis which is also seen in black knot disease progression. Gum formation is triggered by the infection as it causes the glycometabolism to alter, and is accompanied by cell wall degradation (Li et al., 2014). Ethylene (ET) and jasmonic acid are major factors in gum secretion. These are phytohormones commonly elicited under pathogenic conditions, seen by the endogenous application of ET in vitro of peach inoculated with *L. theobromae* resulting in gummosis (Gao et al., 2016).

Quinones have been extensively studied against microbial attacks in plants. Quinone reductases function to protect organisms against oxidative stress (Cheng et al., 2023). In transformed *A. thaliana* plants, quinone oxidoreductase 1 knock out (KO) lines displayed slower lesion growth of necrotrophic fungi, while overexpressed lines were more negatively impacted. The KO lines had higher abundance of ROS which was credited for causing enhanced antifungal reaction (Heyno et al., 2013). *AT3G22550.1* being a NAD(P)H‐quinone oxidoreductase subunit‐2 shuttles electrons from NADPH to quinones in the photosynthetic chain, which control ROS production in the apoplast (Spitsberg & Coscia, 1982). Beyond just killing the pathogen, the trees need to take measures to prevent damage from their own endogenous compounds. This is an important factor in determining genotypes which can withstand black knot attack.

*CRT3* was originally identified in *Plasmodium falciparum* Welch, the malaria causing parasite. Orthologues have since been discovered in *A. thaliana*, *CRT‐like Transporter* (*CLT*), and have been studied for their ability to detoxify and maintain cellular homeostasis. Maughan et al. (2010) reported that *CLT* KO mutants were more prone to *Phytophthora brassicae* (De Cock & Man in ‘t Veld) infection due to altered glutathione availability in the cytoplasm. Consequently, mutants lacking the *CLR* gene showed compromised systemic acquired resistance (SAR). Despite its role as a ROS scavenger, glutathione also serves as a crucial signaling molecule in SAR pathways.

Only one SNP was found in a genic region that resulted in an amino acid change, however it is unconfirmed how and if this alters the gene. SNP20380 was found on chromosome 8, at position 26,070,347, it was an alteration of an adenine to a thymine. This locus is the 5’ UTR of Kelch repeat F‐Box protein, *KFB20*. This gene is responsible for the posttranslational regulation of *PAL* (Zhang et al., 2013). *PAL* is the initial rate limiting step in the phenylpropanoid pathway, which branches off to generate phenolics, flavonoids, anthocyanins and lignins (Pavale et al., 2024). Biotic and abiotic stimuli can illicit the activity of the *PAL* enzyme, which tightly regulates the generation of antimicrobial compounds and protective pigments (Kim & Hwang, 2014). The other side of regulation involves the degradation of *PAL*, which is mediated by the F box proteins (Zhang et al., 2013). Further studies are needed to confirm whether this affects the transcription or translation of the gene.

Our search for candidate genes related to black knot resistance in Japanese plum using the results of our GWAS has been fruitful. We identified eight functional candidate genes which have relationships to plant immunity, biotic stress response and pathogen response. When these genes are dysregulated or inactivated, the plants are far more susceptible to disease, suggesting that these genes are involved in black knot resistance.

## Funding Information

The project was funded by Ontario Ministry of Agriculture, Food and Rural Affairs, Niagara Tender Fruit Marketing Board and Niagara Peninsula Fruit and Vegetable Growers Association to JS.

## CONFLICT OF INTEREST STATEMENT

The authors declared no conflict of interest.

## Supporting information


Data S1.

## Data Availability

All the required data are provided in the manuscript.
