# Supplementary material for: Untying the knot: Unraveling genetic mechanisms behind black knot disease resistance in Prunus salicina (Japanese plum)
Source: Plant Environ Interact. 2024 Nov 5;5(6):e70016. doi: 10.1002/pei3.70016 (PMC11536197; doi:10.1002/pei3.70016)
Supplement: Supplementary file 1 — Data S1. [file PEI3-5-e70016-s001.docx]

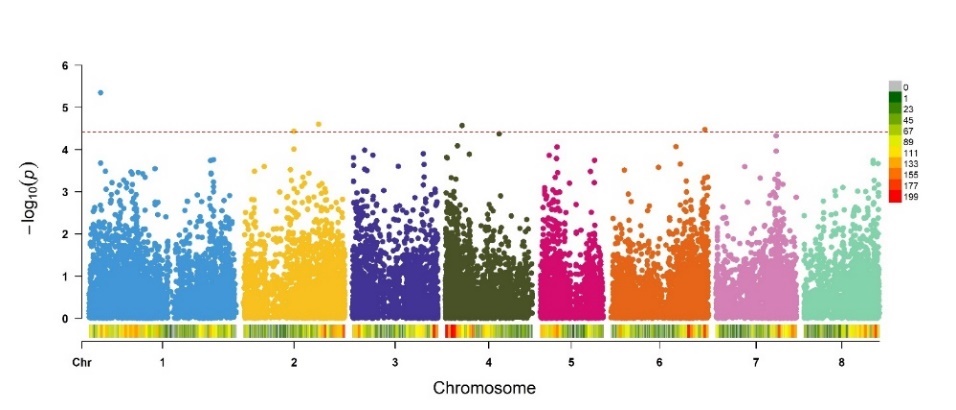

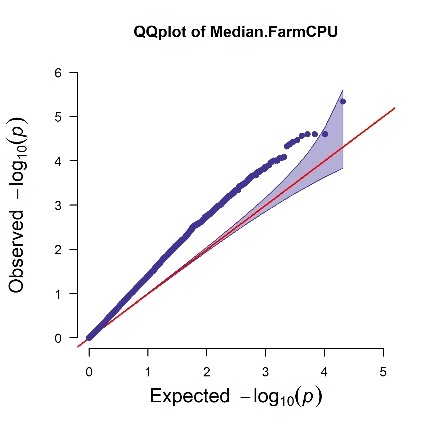

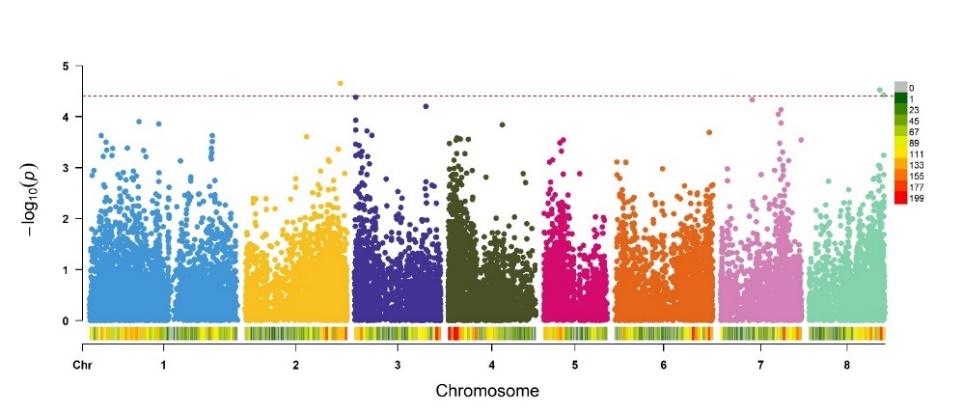

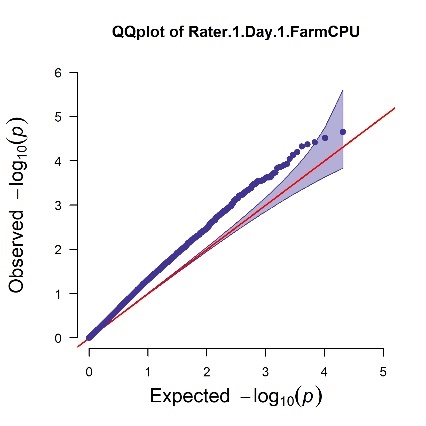

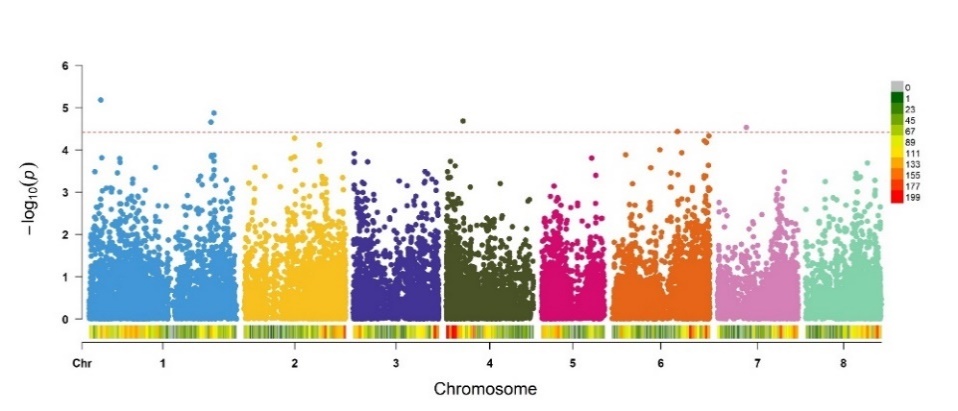

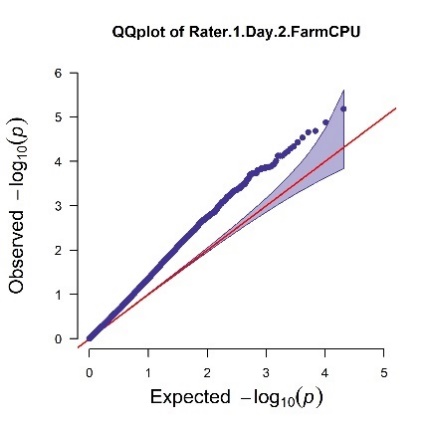

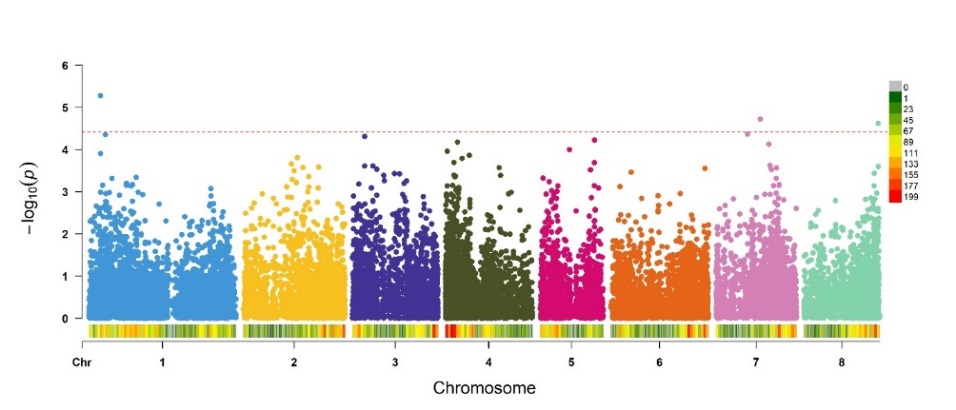

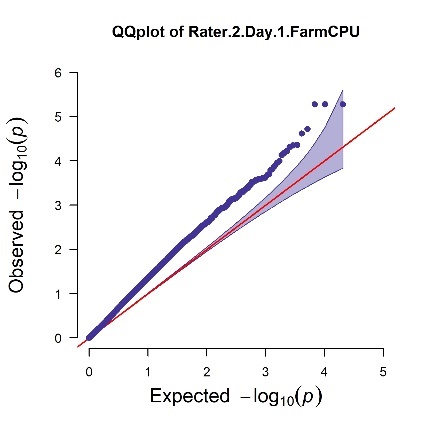


A

D

C

B


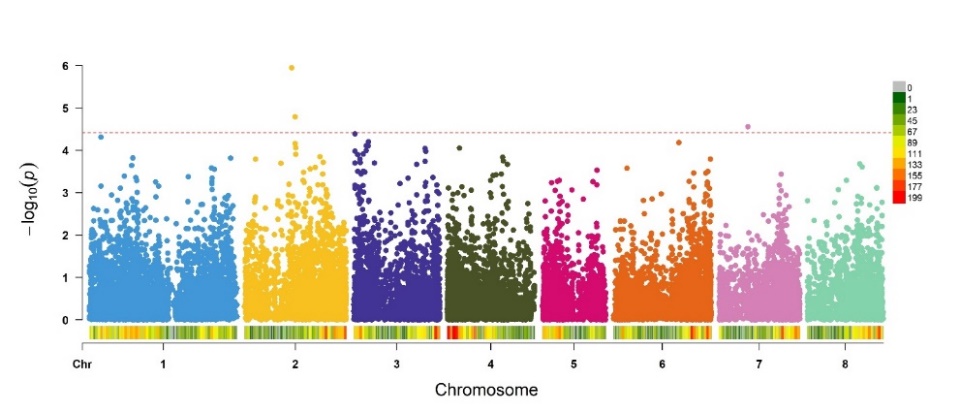

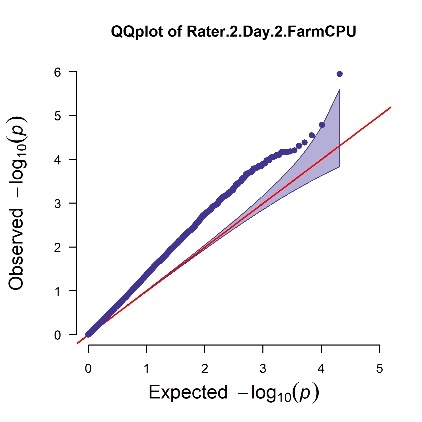


E

Supplementary Figure 1. Manhattan plot using FarmCPU on the right with corresponding quantile-quantile (Q-Q) plot to the left. Colourful bar by the x axis denotes SNP density, legend to the right denotes SNP density increasing as colours become warmer. Red dashed line indicates significance threshold determined by the false discovery rate. a) Median dataset, SNPs can be found on Chromosome 1, 2, 4 and 6. b) Rater 1 Day 1 dataset, SNPs can be found on Chromosome 2 and 8. c) Rater 1 Day 2 dataset, SNPs can be found on Chromosome 1, 4, 6 and 7. d) Rater 2 Day 1 dataset, SNPs can be found on Chromosome 1, 7 and 8. e) Rater 2 Day 2 dataset, SNPs can be found on Chromosome 2, and 7.

| Parental Cross if known, otherwise Genotype Name | Number |
| --- | --- |
| 9904 | 1 |
| 9908 | 2 |
| 9909 | 3 |
| Battema | 4 |
| Burbank x Wickson | 5 |
| Duarte x Wickson | 6 |
| Early Golden | 7 |
| Early Golden x V82051 | 8 |
| Early Golden x Vampire | 9 |
| Gladstone | 10 |
| Morettini 355 | 11 |
| Obil-Naja x Shiro | 12 |
| Obil-Naja x Vanier | 13 |
| Oishi-wase x Vampire | 14 |
| Ouishi-wase | 15 |
| Ozark Premier | 16 |
| Ozark Premier x Vampire | 17 |
| Plumcot | 18 |
| Purple | 19 |
| Redcoat | 20 |
| Redglow | 21 |
| Shiro | 22 |
| Shiro x Vampire | 23 |
| Underwood | 24 |
| V77171 x Shiro | 25 |
| V82011 | 26 |
| V82011 x Early Golden | 27 |
| V82011 x Oishi-wase | 28 |
| V82051 x Early Golden | 29 |
| V82051 x Oishi-wase | 30 |
| V82051 x Ozark premier | 31 |
| V8205 1x Vanier | 32 |
| V82052 x Open | 33 |
| V84021 | 34 |
| V84041 | 35 |
| V84041 x Early Golden | 36 |
| V84041 x Ozark | 37 |
| V85061 | 38 |
| V85061 x Early Golden | 39 |
| V85061 x Shiro | 40 |
| V85061 x Vanier | 41 |
| V85062 | 42 |
| V86011 | 43 |
| V86041 x Open | 44 |
| V86041 x Shiro | 45 |
| V86041 x Vanier | 46 |
| V98031 | 47 |
| V98032 | 48 |
| V98041 | 49 |
| V99021 | 50 |
| Vampire | 51 |
| Vampire x Early Golden | 52 |
| Vampire x Ozark Premier | 53 |
| Vampire x V77171 | 54 |

Supplementary Table 1. A list of the parental crosses corresponding to each number on the PCA plot (Figure 6). Due to space constraints, each genotype was converted into a numerical identifier as seen here.
